# Prevalence of different types of online behavior and internet addiction among adolescents in central siberia: Gender and age aspects

**DOI:** 10.1192/j.eurpsy.2021.996

**Published:** 2021-08-13

**Authors:** N. Semenova, S. Tereshchenko, L. Evert L, Y. Kostyuchenko, M. Shubina

**Affiliations:** 1 Scientific Research Institute For Medical Problems Of The North, Federal Budgetary Scientific Institution «Federal Research Centre «Krasnoyarsk Scientific Centre of Siberian Division of Russian Academy of Sciences», Krasnoyarsk, Russian Federation; 2 Research Institute For Medical Problems In The North, Federal Research Center “Krasnoyarsk Scientific Center of the Siberian Branch of the RAS”, Krasnoyarsk, Russian Federation

**Keywords:** Internet, Addiction, prevalence, Siberia

## Abstract

**Introduction:**

Adolescent online behavior is an urgent public health problem in different countries of the world due to the possibility of developing Internet addiction.

**Objectives:**

To study the prevalence of different types of online behavior and Internet addiction in Siberian adolescents, depending on gender and age.

**Methods:**

During the period from January to May 2019, 2950 adolescents aged 11-18 years living in the urban area of Central Siberia (Krasnoyarsk) were examined, of them 1348 boys and 1602 girls. The Chen Internet Addiction Scale (CIAS) with a cut-off level of 65 points was used. Internet users are divided into three groups: Adaptive Internet Users (AIU) (27–42 points); maladaptive Internet users (MIU) (43–64 points); pathological Internet users (PIU) (score ≥ 65).

**Results:**

The AIU group comprised 50.3% (55.9% boys and 45.6% girls, p <0.001). The share of adolescents aged 11-14 is 52.0%, the share of adolescents aged 15-18 is 48.4% (p = 0.04). The MIU group constituted 42.9% (46.3% were girls and 38.9% boys, p <0.001). The share of adolescents aged 11-14 is 42.1%, and those aged 15-18 is 43.8%, p > 0.05. The PIU group constituted 6.8% (5.1% boys and 8.2% girls, p <0.001). The share of adolescents aged 11-14 is 5.9% and those aged 15-18 is 7.8% (p = 0.04).

**Conclusions:**

Among adolescents in Central Siberia the prevalence of AIU consist 50.3%, MIU 42.9%, PIU 6.8%. The prevalence of PIU is more common in girls. The increase in PIU was marked in the older age group. The study was funded by RFBR project № 18-29-2203218.

**Conflict of interest:**

The reported study was funded by RFBR project № 18-29-22032\18.

